# Forward screening for seedling tolerance to Fe toxicity reveals a polymorphic mutation in ferric chelate reductase in rice

**DOI:** 10.1186/s12284-014-0036-z

**Published:** 2015-01-20

**Authors:** Siriphat Ruengphayak, Vinitchan Ruanjaichon, Chatree Saensuk, Supaporn Phromphan, Somvong Tragoonrung, Ratchanee Kongkachuichai, Apichart Vanavichit

**Affiliations:** Rice Science Center, Kasetsart University, Kamphaengsaen, Nakhon Pathom 73140 Thailand; Interdisciplinary Graduate Program in Genetic Engineering, Kasetsart University, Chatuchak Bangkok, 10900 Thailand; Rice Gene Discovery, National Center for Genetic Engineering and Biotechnology (BIOTEC), National Science and Technology Development Agency (NSTDA), Kasetsart University, Kamphaengsaen Nakhon Pathom, 73140 Thailand; Agronomy Department, Faculty of Agriculture at Kamphaengsaen, Kasetsart University, Kamphaengsaen Nakhon Pathom, 73140 Thailand; Genome Institute, National Center for Genetic Engineering and Biotechnology (BIOTEC), 113 Thailand Science Park, Phahonyothin Road, Khlong Nueng, Khlong Luang, Pathum Thani 12120 Thailand; Institute of Nutrition, Mahidol University, Phutthamonthon 4, Nakhon Pathom, 73170 Thailand

**Keywords:** Rice, Fe-tolerant mutants, Iron toxicity, *OsFRO1*, Fe homeostasis

## Abstract

**Background:**

Rice contains the lowest grain Fe content among cereals. One biological limiting factor is the tolerance of rice to Fe toxicity. Reverse and forward genetic screenings were used to identify tolerance to Fe toxicity in 4,500 M_4_ lines irradiated by fast neutrons (FN).

**Findings:**

Fe-tolerant mutants were successfully isolated. In the forward screen, we selected five highly tolerant and four highly intolerant mutants based on the response of seedlings to 300 ppm Fe. Reverse screening based on the polymorphic coding sequence of seven Fe homeostatic genes detected by denaturing high performance liquid chromatography (dHPLC) revealed MuFRO1, a mutant for *OsFRO1* (LOC_Os04g36720). The MuFRO1 mutant tolerated Fe toxicity in the vegetative stage and had 21-30% more grain Fe content than its wild type. All five highly Fe-tolerant mutants have the same haplotype as the MuFRO1, confirming the important role of *OsFRO1* in Fe homeostasis in rice.

**Conclusions:**

FN radiation generated extreme Fe-tolerant mutants capable of tolerating different levels of Fe toxicity in the lowland rice environment. Mutants from both reverse and forward screens suggested a role for *OsFRO1* in seedling tolerance to Fe toxicity. The MuFRO1 mutant could facilitate rice production in the high-Fe soil found in Southeast Asia.

**Electronic supplementary material:**

The online version of this article (doi:10.1186/s12284-014-0036-z) contains supplementary material, which is available to authorized users.

## Findings

### Fe toxicity tolerance and grain Fe content

Fe toxicity is a serious agricultural problem, particularly when plants are grown in acidic soils (Quinet et al. 2012). More than 100 million hectares of lowland rice production on low-pH soil in Southeast Asia is limited by iron toxicity (Becker and Asch 2005). Fe toxicity can occur in flooded soils with a pH below 5.8 under aerobic conditions, and at a pH below 6.5 under anaerobic conditions (Fageria et al. 2008). Plants grown under such conditions accumulated two-fold more Fe in their leaves (Bashir et al. 2014). In low pH paddy field, anaerobic condition leads to the reduction of Fe^3+^ to Fe^2+^, resulting in excessive Fe availability and increased absorption (Quinet et al. 2012).

Genetic variation for tolerance to Fe toxicity exists in local landraces. However, most of their adaptive mechanism is associated with genetic variation in avoidance to Fe absorption and resulting in low grain Fe density. That association limits the chance for improving grain Fe density in acid soil, where high levels of Fe^+2^ are available for uptake and translocation to the grain. Therefore, it is important to understand natural genetic variation in enriching grain Fe density under Fe toxicity. One of the tolerance mechanisms that reduce excess Fe absorption is by reducing Fe^+2^ concentrations in rhizosphere by increasing the oxidative capability of roots (Ando 1983) or by excluding Fe from the rhizosphere (Tadano 1976). Another possible mechanism is increasing tissue tolerance to excessive levels of Fe^+2^ while increasing the rate of mobilization to grains. Such tolerance rice may link to Fe homeostasis that is not easily identified in existing germplasm. Therefore, one strategy is to find double mutation combining moderate-to-high grain Fe content under neutral pH soil conditions while maintaining in the ability to withstand Fe-toxic conditions. These mutants may be likely to gain more Fe^+2^ to transport excessive Fe to grains.

### FN mutant library

**Figure 1:**
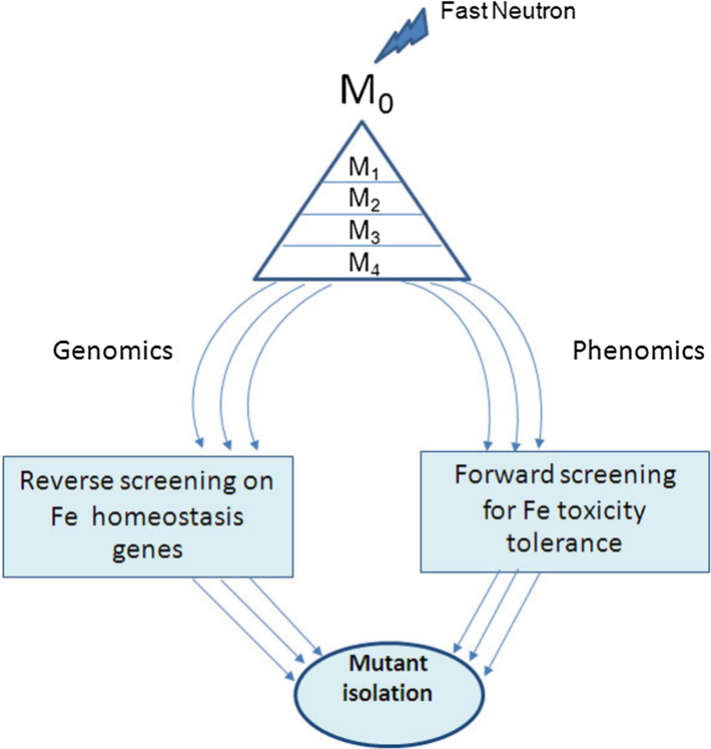
**Schematic view of mutant discovery for rice mutant tolerance to Fe toxicity by reverse and forward genetic screens of a large FN-treated population.**

The fast neutron library was developed from Jao Hom Nin (JHN), a photoperiod non-sensitive, purple rice variety. By taking advantages of its distinctive color of leaves and grains, semi-dwarfism, early flowering and nutrient- rich grains, such mutant population is valuable for discovering mutation expressing useful genetic variation for both agronomic and nutritive characteristics. With its high combining ability, JHN mutants could be utilized as sources of new traits for marker-assisted selection in rice. Approximately 100,000 breeder seeds from JHN were mutagenized by using 33 Gy fast neutrons (FN). Successive generations from M_1_-M_4_, family history was traceable from individual M_1_ plant. Due to abnormal mutation affecting seed set, several families were terminated leaving only 21,024 M_4_ mutant families forming the base population for genetic screening (Rice Science Center, Kasetsart University, Thailand). For Fe toxicity screening, 4,500 lines were randomly chosen for forward screening while 500 pooled DNA libraries (representing 24 M_1_ plants per pool) from the M_4_ generation were used for reverse screening (Figure 1).

### Forward screening identified Fe toxicity tolerant mutants

**Figure 2:**
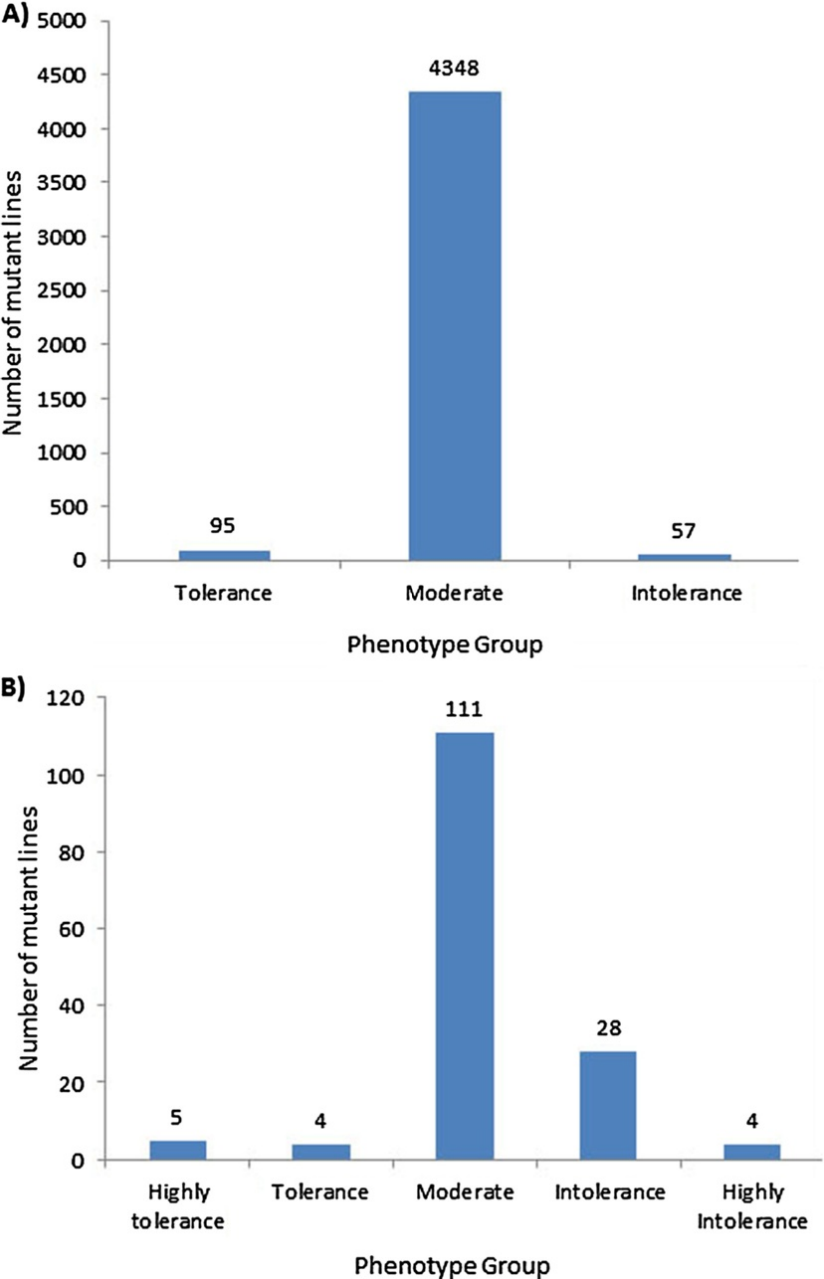
**Distribution of responses to Fe toxicity of 4,500 M**_**4**_ **lines in Fe-toxic (pH 3.0, FeEDTA 300 ppm) nutrient solution at the five-leaf seedling stage.** The outcome of **A)** the 1st round of screening and **B)** the 2nd round of screening on 152 M_4_ mutants consisting of 95 tolerant and 57 intolerant M_4_ lines selected from the first round of mutants.

**Figure 3:**
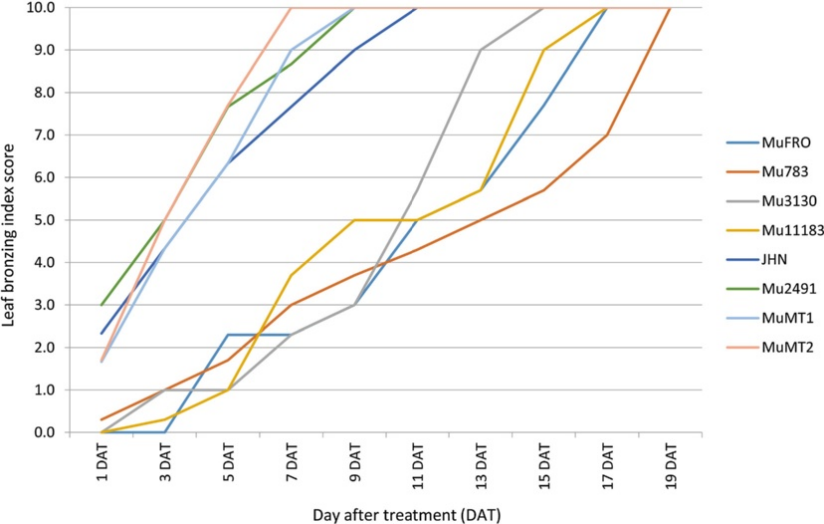
**Average leaf bronzing index (LBI) scored after exposure to Fe toxicity on seven selected mutants compared with the JHN wild type.** (DAT: Day after treatment).

The 4,500 lines were screened in Fe-toxic (pH 3.0, FeEDTA 300 ppm) nutrient solution at the five-leaf seedling stage. The low pH nutrient solution released excessive ferrous Fe^+2^, resulting in extensive leaf bronzing. The base population was assessed using a leaf bronzing index (LBI) (Arbeit 2003) in a time-course manner following the death of the wild type JHN seedlings. In addition, the localization of iron in various parts of the leaf was visualized by PPB staining (Prom-U-thai et al. 2003). The days-to-seedling-death (DSD) of each variety was also recorded every other day. Phenotypic responses to Fe toxicity were categorized into tolerant, moderate and intolerant based on LBI and leaf PPB. Rice grains were also stained with PPB to reveal the embryonic Fe content, which is strongly associated with the grain Fe density. The first round screening of the 4,500 M_4_ families yielded 95 tolerant and 57 intolerant mutants (Figure 2A). After repeated screenings, only 9 tolerant and 32 intolerant lines remained (Figure 2B). Selected mutants were scored for LBI every other day under the Fe toxic treatment (Figure 3). The result showed that the LBI of each mutant line increased rapidly for 20 days after Fe toxicity treatment, but the rate of increase can clearly be divided into tolerant and intolerant groups. By the 5th day, some intolerant mutant lines began to die, while the tolerant lines by the 11th day. The tolerant Mu783 prolonged seedling death to 19 days.

### Designing the reverse screen

Mutant lines identified by forward screening can be used to develop functional markers for marker-assisted breeding. However, gene identification via forward mutant screen is complex, as FN-induced mutagenesis hits multiple targets. By reverse genetics, putative mutants carrying the candidate allele can be directly screened for the target phenotypic changes. This approach is called TILLING, Targeted Induced Local Lesion in Genome (Till et al. 2003). Using TILLING, seven candidate genes for iron homeostasis were selected, including *ferric chelate reductase1 (OsFRO1;* [MSU: LOC_Os04g36720]*), ferritin 1 (OsFer1;* [MSU: LOC_Os11g01530])*, ferritin 2 (OsFer2;* [MSU: LOC_Os12g01530]), *iron regulated transporter 1 (OsIRT1;* [MSU: LOC_Os03g46470]), *nicotianamine synthase 3 (OsNAS3;* [MSU: LOC_Os07g48980]), *frataxin (OsFx*; [MSU: LOC_Os01g57460]) and *yellow stripe leaf 16 (OsYSL16*; [MSU: LOC_Os04g45900]) (Gross et al. 2003 and Kawahara et al. 2013) for reverse screening using Denaturing High Performance Liquid Chromatography (DHPLC).

Gene-specific primers were designed for polymorphic coding sequences of the seven candidate genes for reverse screening*.* Potential mutable sequence variations were identified for each candidate gene and queried for potential SNV via public domains. Selected differential genotypes for grain Fe density, including Xua Bue Nuo (XBN), JHN, IR68144, RB#3, KDML105 (KD), Azucena (Azu) and Nipponbare, were genotyped for the SNVs, which may be associated with tolerance to Fe toxicity (Additional file 1: Table S1). Primer pairs for PCR amplification and denaturing conditions of each mutable site for dHPLC are listed (Additional file 2: Table S2). Before injection into the dHPLC column, PCR amplicons were denatured at 95°C for 5 min and annealed gradually from 95°C to 65°C over 30 min (Callery et al. 2006).

### Reverse screen by heteroduplex

**Figure 4:**
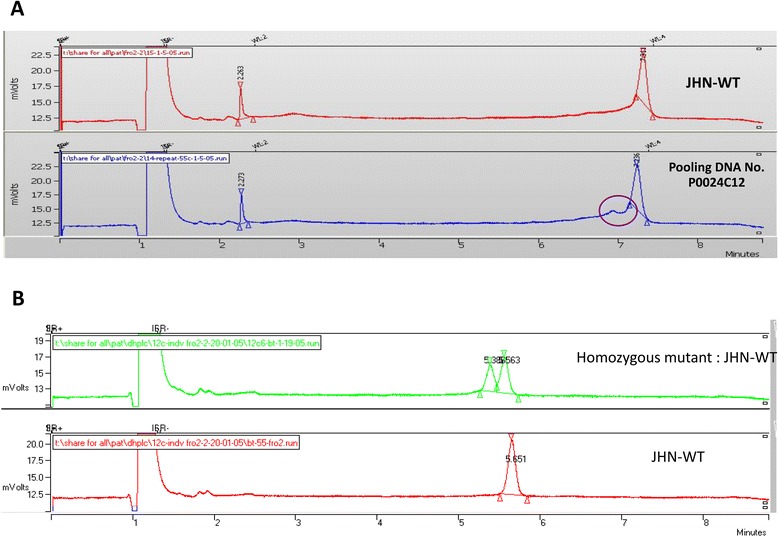
**The dHPLC chromatograms of the** ***OsFRO1*** **amplicons. A)** The heteroduplex chromatogram was identified on 1D-DNA pooling No. P0024C12 (ratio1:24) compared to JHN-WT. **B)** The individual mutant line, a member of DNA pool No. P0024C12 that contained the mutant genotype, was identified in a 1:1 admixture with JHN WT.

Reverse screen was initiated among 192 M_4_ genomic DNA pools. Each pool represented either a set of 24 M_1_ lines, or the total 4,608 lines. Amplified target fragments from the six candidate genes were screened for heteroduplexes using dHPLC in a 1:1 admixture with the control (wild type) amplicons. The results indicated that heteroduplex was only detected on *OsFRO1* amplicons in DNA pool no.P024C12 (Figure 4A). Individual members of the P024C12 pool were analyzed for potential mutants. Heteroduplex-forming amplicons were confirmed by sequencing (Figure 4B). No mutation was found in the remaining candidate genes. However, we cannot rule out the possibility of mutations within other parts of the candidate genes, such as introns and promoters that were not included in the design.

### Mutation on *OsFRO1*

The *OsFRO1* mutant was purified and designated ‘MuFRO1’. The *OsFRO1* target gene was confirmed by gene sequencing. Sequence comparison between wild type and MuFRO1 identified four new single nucleotide polymorphisms (SNPs) and one indel in several introns. Two single amino acid changes (SAP) were identified in Exons 4 and 5 (Table 1). One SAP on Exon 4 exhibited a Valine (V) to Isoleucine (I) change in the same hydrophobic group. Because the SAP is located within the ferric chelate reductase domain, the amino acid change may affect the functioning of *OsFRO1* (Marchler-Bauer et al. 2013). *OsFRO1* also contained an AAA deletion, a SNP in intron 2 and three SNPs in intron 3, similar to the mutations found in KDML105, the landrace Fe toxicity tolerant strain, but unlike JHN and IR68144, the intolerant varieties (Table 1). Therefore, there are multiple FN-induced SNVs in the mutant *OsFRO1*. We cannot rule out the possibility of finding more mutation on other part of the genome.

**Table 1:** **Haplotype variation of** ***OsFRO1*** **in MuFRO1 compared to tolerant (KDML105) and intolerant (IR68144) controls**

**Location on gene structure**	**Position on MSU7**	**Rice varieties**				**OryzaSNP @MSU ID**
		**JHN**	**MuFRO1**	**IR68144**	**KDML105**	
Intron2	Between 22183415&22183416	AAA	-	AAA	-	-
Intron2	22183525	G	A	G	A	TBGI203991
Intron3	22183880	A	G	A	G	-
Intron3	22183900	C	T	C	T	-
Intron3	22184066	C	T	C	T	-
**Exon4 (V > I)**	22184296	**G**	**A**	**G**	**A**	**-**
**Exon5 (S > C)**	22184982	**C**	**G**	**C**	**G**	TBGI203998

Ferric chelate reductase was first reported in Arabidopsis (Robinson et al. 1999) and later two *FRO*-like genes were identified in rice (Ishimaru et al. 2006). *OsFRO1* was detected in leaves of Zn, Mn and Cu deficient rice whereas *OsFRO2* transcript was found on Fe-deficient leaves but not in roots under Fe deficiency. The result indicated that rice posses a unique Fe^2+^- uptake system via *OsIRT1* and *OsIRT2*. A transgenic plant that fused *refre1/372* from high pH tolerant yeast with the promoter of *OsIRT1* showed strong increase in Fe^3+^ chelate-reductase activity and Fe-uptake rate than control under Fe-deficient conditions. When grown under calcareous soil with high pH and low Fe availability, the transgenic rice yielded 7.9 times more productive. Recently, subcellular localization of the FRO families from Arabidopsis were identified (Jain et al. 2014). *AtFRO7*, found in chloroplast, may play important roles in Fe transport into chloroplast whereas *AtFRO3* and *AtFRO8,* found in mitochondria, may involve in mitochondrial metal ion homeostasis (Jain et al. 2014). Cellular function of *OsFRO2* was recently identified as a membrane bound protein in mitochondria (Emanuelsson et al. 2000). However, no report concerns the exact localization of *OsFRO1* in rice and its role in Fe trafficking under Fe toxic conditions. Recently, transcriptomic analysis of rice grown under contrasting Fe levels revealed *OsFRO2* was up-regulated under Fe deficiency but reverse under excessive Fe in shoots (Bashir et al. 2014). Furthermore, under Fe toxic condition, peroxidases, the enzyme known to cope with reactive oxygen species (ROS), was up-regulated in root (Quinet et al. 2012). Comparison between rice varieties with high and low grain Fe density, *OsFRO1* expression in grains show no difference (Das et al. 2013). However, only *OsFRO1* transcript was found in root of the high grain Fe density variety. This finding leads to more investigation on the role of *OSFRO1* in enriching grain Fe content for rice grown under excessive Fe.

### Development of functional markers

**Figure 5:**
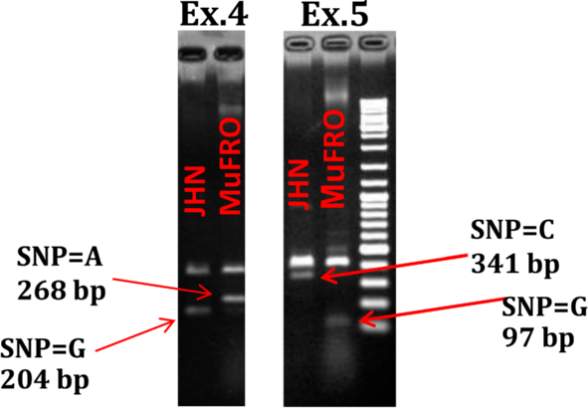
**Genotyping of JHN and MuFRO1 using bi-directional SNP primers for a single amino acid polymorphism (SAP) in Exons 4 and 5.** Expected amplicon size of the SAP in Exon 4: SAPEx.4 F/R = 435 bp, SAP_A/SAPEx. 4_R = 268 bp and SAPEx.4 F/SAP-G = 204 bp. Expected amplicon size of the SAP in Exon 5: SAPEx.5 F/R = 402 bp, SAPEx.5 F/SAP-C = 341 bp and SAP_G/SAPEx. 5_R = 97 bp.

Marker-assisted selection is most efficient when the functional marker for a target trait is utilized. For Fe toxicity tolerance, the two non-synonymous SAPs on Exons 4 and 5 of the *OsFRO1* gene are used as functional markers. To develop agarose-based, co-dominant markers for Fe toxicity tolerance, bi-directional PCR was developed (Table 2), combining four primers in a single PCR amplification (Liu et al. 1997). Target amplicons were detected by 1.2% agarose gel electrophoresis (Figure 5). These primer set and amplification protocols can be used for marker-assisted selection to improve tolerance to Fe toxicity.

**Table 2:** **Bi-directional SNP primer sequences of** ***OsFRO1***

Primer name*	**Sequence 5’ → 3’**
OsFRO1_Ex.4F	GGTGGATGAAGACACTACTGC
OsFRO1_Ex.4R	CACAGGACATTGGTCATAGCA
OsFRO1_Ex.4_SAP_A	GGCCTCCGGTTCGGATCGA
OsFRO1_Ex.4_SAP_G	GCCATGCAAAACAACCCGAC
OsFRO1_Ex.5F	TCATCTACTCTGTTTTGGAGGT
OsFRO1_Ex.5R	CTTGCTGGCTTTGAGAAGACT
OsFRO1_Ex.5_SAP_G	TTCCTGAGGTTCTGGCAATG
OsFRO1_Ex.5_SAP_C	TGTCCACCTTGGCCCTGG

*Each SAP primer set was amplified using the KAPA 2 G Robust HS protocol (KAPABIOSYSTEMS, Woburn, USA) under the following thermal cycling conditions: one cycle at 95°C for 3 min; 35 cycles of 30 s denaturation at 95°C, 30 s annealing at 61°C, and 30 s extension at 72°C; and a final extension at 72°C for 2 min.

Two haplotypes of the two SAPs, ‘A-G’ and ‘G-C’, can differentiate the highly tolerant samples from the rest (Table 3). Genotyping of the 41 selected mutants for forward screening (Figure 2B) revealed four new highly Fe toxicity-tolerant mutants, Mu1463, Mu3130, Mu11183 and Mu783. Phenotypic screening confirmed mutants that were highly tolerant (5), moderate (1) and highly intolerant (4) to Fe toxicity. Such haplotypes can be directly applied to MAS for Fe toxicity tolerance.

**Table 3:** ***OsFRO1*** **haplotypes and grain PPB scores on Fe-tolerant mutants and standard rice cultivars**

**Variety**	***OsFRO1*** **Haplotype**	**Phenotype**	
		**PPB score**	**Fe toxicity**
MuFRO1	*OsFRO1* ^A-G^	++	Highly tolerant
Mu1463	*OsFRO1* ^A-G^	+	Highly tolerant
Mu3130	*OsFRO1* ^A-G^	+	Highly tolerant
Mu11183	*OsFRO1* ^A-G^	+	Highly tolerant
Mu783	*OsFRO1* ^A-G^	+	Highly tolerant
Mu2409	*OsFRO1* ^G-C^	+	Tolerant
Mu2559	*OsFRO1* ^G-C^	+	Tolerant
Mu2638	*OsFRO1* ^G-C^	+	Tolerant
Mu3113	*OsFRO1* ^G-C^	+	Tolerant
JHN	*OsFRO1* ^G-C^	+	Moderate
MuMT1	*OsFRO1* ^G-C^	++	Highly intolerant
MuMT2	*OsFRO1* ^G-C^	++	Highly intolerant
Mu2491	*OsFRO1* ^G-C^	+	Highly intolerant
Mu3295	*OsFRO1* ^G-C^	+	Highly intolerant
IR68144	*OsFRO1* ^G-C^	++	Highly intolerant
KDML105	*OsFRO1* ^A-G^	0	Highly tolerant
Pinkaset#3	*OsFRO1* ^G-C^	0	Tolerant
RIL 909-21-2-5	*OsFRO1* ^A-G^	0	Highly tolerant

In fast neutron treated population, it is not uncommon to identify multiple mutated genes from reverse screen. To ascertain if the multiple gene mutation was not false positive, we conducted a whole genome sequencing of the wild type JHN and extensive GBS of selected mutated genes using random lines core collection of the JHN mutant population to see if there have already existed in the JHN.

### Target sequencing on MuFRO1

To further investigate the possibility of finding more candidate genes, targeted enrichment sequencing was conducted on 40 candidate genes that play roles in Fe transport from soil to seeds (Gross et al. 2003; Koike et al. 2004; Bashir et al. 2010; Masuda et al. 2012). The nucleotide sequence of 40 candidate genes (240 Kb) was collected for probe design (Additional file 3: Table S3). Targeted enrichment sequencing was conducted based on the Sure Select-XT Target enrichment system (Illumina paired end and multiplexed sequencing library by Agilent Technologies).

A new missense nucleotide variation was identified in *OsYSL7*, the metal-nicotinamide transporter protein (Table 4). The expression of *OsYSL5*, *OsYSL6*, *OsYSL7*, *OsYSL14* and *OsYSL17* were detected in epidermis, exodermis, cortex and stele of 3 week-old seedling root grown under Fe-deficient conditions for 2 weeks (Inoue et al. 2009). While, no expression was detected from maximum tillering to the flowering stages (Chandel et al. 2010).

**Table 4:** **Single nucleotide variant (SNV) for type, the location and/or effect of each SNV on MuFRO1 compared with JHN**

**LOC**	**Gene symbol**	**Chro.**	**Position on MSU7**	**MuFRO1**	**JHN-WT**	**SNP classification**	**Known SNVs**
LOC_Os02g02450	*OsYSL7*	2	863203	C	T	Missense variant (S > L)	rs18774481
LOC_Os03g46470	*OsIRT1*	3	26284263	-	C	Intron	-
LOC_Os04g36720	*OsFRO1*	4	Between 22183415&22183416	-	AAA	Intron	-
LOC_Os04g36720	*OsFRO1*	4	22183525	A	G	Intron	TBGI203991
LOC_Os04g36720	*OsFRO1*	4	22183880	G	A	Intron	-
LOC_Os04g36720	*OsFRO1*	4	22183900	T	C	Intron	-
LOC_Os04g36720	*OsFRO1*	4	22184066	T	C	Intron	-
LOC_Os04g36720	*OsFRO1*	4	22184296	A	G	Missense variant	-
LOC_Os04g36720	*OsFRO1*	4	22184982	G	C	Missense variant	TBGI203998

All sequence variations were compared to reported SNVs in the Gramene (rs) and OryzaSNP (TBGI) databases.

### Phenotypic evaluation of MuFRO1

**Figure 6:**
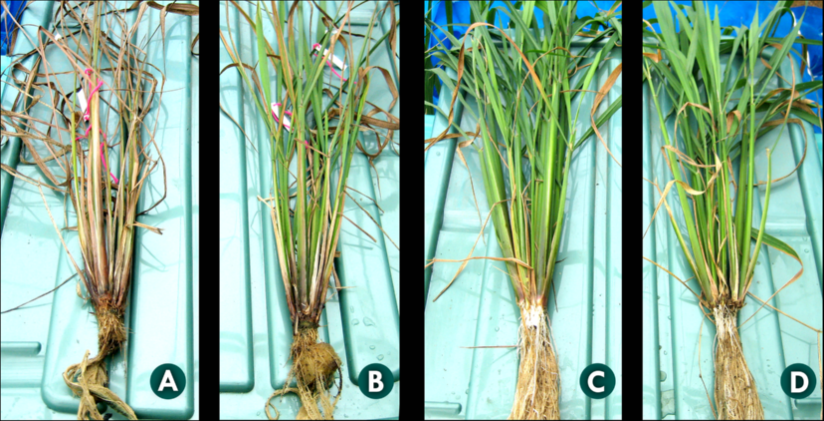
**Rice plant treated with toxic nutrient solutions (300 ppm) for three weeks: A) wild type and B) MuFRO1 and under control (4 ppm) conditions: C) wild type and D) MuFRO1.**

Seeds of JHN and MuFRO1 harvested from two planting seasons were analyzed for Fe, Zn and Cu contents using ICP-OES at the Institute of Nutrition, Mahidol University, Thailand. The Fe toxicity hydroponic experiment was conducted to evaluate the effects of *OsFRO1* on iron homeostasis. Five seedlings of MuFRO1 and JHN at the tillering stage were grown in normal (pH 5.5, FeEDTA 4 ppm) and toxic (pH 3.0, FeEDTA 300 ppm) levels of Fe nutrient solution (Yoshida et al. 1976) for three weeks. The total Fe concentration in shoots was compared between MuFRO1 and JHN. The results indicated that under control conditions, MuFRO1 and JHN wild type contains 64.44 ± 2.59 ppm and 27.29 ± 0.70 ppm of total shoot Fe, respectively, or 136% higher than JHN whereas no substantial difference on their dry weights of the two samples. On the other hand, under Fe toxic conditions, the Fe concentrations in shoot of MuFRO1 and JHN were 639.69 ± 10.76 ppm and 847.85 ± 21.71 ppm, respectively (Table 5). MuFRO1 remained green with more biomass (data not shown) than wild type (Figure 6). This result may suggest that MuFRO1 performed better Fe homeostasis by maintaining lower Fe content in the shoots. One such mechanism is simply by efficient partitioning into storage organelles like mitochondria and chloroplast. Therefore, *OsFRO1* may play important roles in iron homeostasis and the maintenance of high biomass when grown under Fe toxicity conditions. JHN and MuFRO1 seeds were analyzed for Fe and Zn contents. MuFRO1 seeds contained 30% more grain iron than wild type JHN, but there was no difference in zinc content (Table 6). This opening a new opportunity to develop new rice varieties to withstand lowland Fe toxicity as well as enrichment of grain Fe density in the greater lowland rice growing area in the rice bowl of Asia.

**Table 5:** **Fe contents (ppm) of JHN stem and leaf compared with MuFRO1 grown under control (4 ppm Fe) and Fe-toxic (300 ppm Fe) nutrient conditions**

**Variety**	**Control**			**Toxic**		
	**Stem**	**Leaves**	**Total in shoot**	**Stem**	**Leaves**	**Total in shoot**
JHN	9.57 ± 0.92	17.72 ± 0.64	27.29 ± 0.70	476.17 ± 9.20	371.68 ± 9.21	847.85 ± 21.71
MuFRO1	35.52 ± 1.15	28.92 ± 1.99	64.44 ± 2.59	204.18 ± 6.11	435.51 ± 5.32	639.69 ± 10.76

**Table 6:** **Iron and zinc contents in JHN and MuFRO1 brown rice seeds from two generations grown in normal soil conditions**

**No.**	**Variety**	**Fe (ppm)**	**Zn (ppm)**
1	MuFRO1 (M_4_ seed)	13.7 ± 0.38	17.7 ± 0.35
2	JHN (control)	10.5 ± 0.44	16.6 ± 0.65
3	MuFRO1 (M_5_ seed)	11.7 ± 0.30	25.8 ± 1.07
4	JHN (control)	9.4 ± 0.25	25.4 ± 0.56

## Additional files

Additional file 1: Table S1. Natural sequence variation on two *ferritin* gene (*OsFer1* and *OsFer2*) and *Ferric chelate reductase1* (*OsFRO1*) among selected varieties that differ in iron density. Additional file 2: Table S2. Primer pairs and denaturing conditions for each mutable site. Additional file 3: Table S3. Positions of 40 candidate genes (240 Kb) involving iron uptake, transport and storage in rice.

## Abbreviations

AAS: Atomic Absorption Spectroscopy
DHPLC: Denaturing High Performance Liquid Chromatography
FN: Fast neutrons
ICP-OES: Inductively coupled optical emission spectrometry
JHN: Joa Hom Nin
LBI: Leaf bronzing index
MuFRO1: *Ferric chelate reductase* mutant
PPB: Perl Prussian Blue
SAP: Single amino acid polymorphism
SNP: Single nucleotide polymorphism
SNV: Single nucleotide variant
TILLING: Targeting Induced Local Lesions IN Genomes
