# Supplementary material for: Forward screening for seedling tolerance to Fe toxicity reveals a polymorphic mutation in ferric chelate reductase in rice
Source: Rice (N Y). 2015 Jan 20;8:3. doi: 10.1186/s12284-014-0036-z (PMC4883132; doi:10.1186/s12284-014-0036-z)
Supplement: Additional file 1: Table S1. — Natural sequence variation on two ferritin gene (OsFer1 and OsFer2) and Ferric chelate reductase1 (OsFRO1) among selected varieties that differ in iron density. [file 12284_2014_36_MOESM1_ESM.doc]

**Supplementary table S1:** Natural sequence variation on two *ferritin* gene (*OsFer1* and *OsFer2*) and *Ferric chelate reductase1* (*OsFRO1*) among selected varieties that differ in iron density.

| Gene | Location on gene structure | Rice varieties | | | | | | |
| --- | --- | --- | --- | --- | --- | --- | --- | --- |
|  |  | Intermediate to high grain iron | | | | Low grain iron | | |
|  |  | JHN | XBN | IR68144 | RB#3 | KD | Azu | Nip |
| *OsFer1* | Upstream | A | A | A | A | G | G | G |
|  | Upstream | T | T | T | T | C | C | C |
|  | Upstream | G | G | G | G | A | A | A |
|  | Upstream | G | G | G | G | A | A | A |
|  | Upstream | G | G | G | G | A | A | A |
|  | Upstream | ATATATAGAT | ATATATAGAT | ATATATAGAT | ATATATAGAT | - | - | - |
|  | Upstream | C | C | C | C | T | T | T |
|  | Upstream | G | G | G | G | A | A | A |
|  | Upstream | G | G | G | G | A | A | A |
|  | Upstream | A | A | A | A | G | G | G |
|  | Upstream | T | T | T | T | G | G | G |
|  | Upstream | G | G | G | G | C | C | C |
|  | Upstream | C | C | C | C | A | A | A |
|  | Exon 2 (Synonymous) | G | G | G | G | A | A | A |
|  | Downstream | G | G | G | G | C | C | C |
|  | Downstream | T | T | T | T | - | - | - |
|  | Downstream | A | A | A | A | G | G | G |
|  | Downstream | G | G | G | G | A | A | A |
|  | Downstream | A | A | A | A | C | C | C |
| *OsFer2* | Upstream | G | nd* | C | nd | G | nd | C |
|  | Upstream | C | G | G | C | C | G | G |
|  | Exon 1 | C | nd | G | nd | C | nd | G |
|  | (Synonymous) |  |  |  |  |  |  |  |
|  | Downstream | A | A | G | A | A | G | G |
|  | Downstream | C | C/T | T | C/T | C | T | T |
|  | Downstream | C | C | G | C | C | G | G |
|  | Downstream | A | A | G | A | A | G | G |
| *OsFRO1* | Intron 2 | AAA | nd | AAA | nd | - | nd | - |
|  | Intron 2 | G | nd | G | nd | A | nd | A |
|  | Intron 3 | A | nd | A | nd | G | nd | G |
|  | Intron 3 | C | nd | C | nd | T | nd | T |
|  | Intron 3 | C | nd | C | nd | T | nd | T |
|  | Exon 4 (V>I) | G | nd | G | nd | A | nd | A |
|  | Exon 5 (S>C) | C | nd | C | nd | G | nd | G |

nd*: not studied
