# Supplementary material for: Forward screening for seedling tolerance to Fe toxicity reveals a polymorphic mutation in ferric chelate reductase in rice
Source: Rice (N Y). 2015 Jan 20;8:3. doi: 10.1186/s12284-014-0036-z (PMC4883132; doi:10.1186/s12284-014-0036-z)
Supplement: Additional file 2: Table S2. — Primer pairs and denaturing conditions for each mutable site. [file 12284_2014_36_MOESM2_ESM.doc]

**Supplementary table S2:** Primer pairs and denaturing conditions for each mutable site.

| **LOC** | **Gene name** | **Primer name** | **Forward primer** (5’---3’) | **Reverse primer** (5’---3’) | **Amplicon Size** | **DHPLC melt. temp (°C)** |
| --- | --- | --- | --- | --- | --- | --- |
| LOC_Os04g36720 | *OsFRO1* | FRO1_2 | GGGAGTATTTTCCTCGCATTC | TGGCCAAATTTTGAGATGAG | 664 | 61 |
| LOC_Os11g01530 | *OsFer1* | Fer1_6  Fer1_pro | AAGAAGAAATGCGCACATGA  GGAGGATATTTTGGGCTGGT | CCACCGCACAATTAATCTCA  CCATCAGCTTGCAAGGAAAG | 436  318 | 65  55 |
| LOC_Os12g01530 | *OsFer2* | Fer2_Ex.1  Fer2_Ex2-3 | CCAAACATCGGACTAAAGAGG  GGCGTAAGCAGAAGCACAAG | CACCGCACAATCTCATCACT  CGATATGAAGTCCCTGTTCCA | 759  591 | 59  60 |
| LOC_Os03g46470 | *OsIRT1* | IRT1_2 | TGAAACAATATGGTCGGTCA | TCTGCAGCTGATGATCGAGT | 637 | 59 |
| LOC_Os07g48980 | *OsNAS3* | NAS3_3 | CCGCCTCCTTCCACAACTA | TACGAGGAGGGCAGCTTCT | 539 | 66 |
| LOC_Os04g45900 | *OsYSL16* | *YSL16*_2 | GACGTCGAACGCCTTGTAGA | GCCTCGCGTATTTTCAGTTC | 559 | 64 |
| LOC_Os01g57460 | *OsFx* | Fx_1 | GGGTTAACGCATCCTCCTC | TCGAACTTTGTGCTGAACCA | 628 | 60 |
