# Supplementary material for: Forward screening for seedling tolerance to Fe toxicity reveals a polymorphic mutation in ferric chelate reductase in rice
Source: Rice (N Y). 2015 Jan 20;8:3. doi: 10.1186/s12284-014-0036-z (PMC4883132; doi:10.1186/s12284-014-0036-z)
Supplement: Additional file 3: Table S3. — Positions of 40 candidate genes (240 Kb) involving iron uptake, transport and storage in rice. [file 12284_2014_36_MOESM3_ESM.doc]

**Supplementary table S3:** Positions of 40 candidate genes (240 Kb) involving iron uptake, transport and storage in rice.

| **No.** | **gene locus name** | **Chr** | **Start** | **End** | **Region of Interest ROI (bp)** | **Description** | **Gene Symbol** | **accession number** |
| --- | --- | --- | --- | --- | --- | --- | --- | --- |
| 1 | LOC_Os01g31870 | 1 | 17451977 | 17465003 | 13026 | natural resistance-associated macrophage protein, putative, expressed | *OsNramp6(TM11)* | NM_001049674 |
| 2 | LOC_Os01g42380 | 1 | 24073021 | 24082137 | 9116 | pleiotropic drug resistance protein, putative, expressed-ABC transporter | *OsPDR9(TM13)* | NM_001050074 |
| 3 | LOC_Os01g61390 | 1 | 35499807 | 35505009 | 5202 | transposon protein, putative, unclassified, expressed | *OsYSL18(TM 12)* | NM_001051227 |
| 4 | LOC_Os01g74110 | 1 | 42903522 | 42907430 | 3908 | metal cation transporter, putative, expressed | *OsZIP1(TM 9)* | NM_001052075 |
| 5 | LOC_Os02g02450 | 2 | 861272 | 866065 | 4793 | transposon protein, putative, unclassified, expressed | *OsYSL7(TM11)* | NM_001052212 |
| 6 | LOC_Os02g02460 | 2 | 865007 | 870292 | 5285 | transposon protein, putative, unclassified, expressed | *OsYSL8(TM 13)* | NM_001052213 |
| 7 | LOC_Os02g03900 | 2 | 1657576 | 1663644 | 6068 | metal transporter Nramp6, putative, expressed | *OsNramp4(TM12)* | NM_001052329 |
| 8 | LOC_Os02g20360 | 2 | 11996090 | 12003629 | 7539 | tyrosine aminotransferase, putative, expressed | *OsNAAT1* | NM_004952265 |
| 9 | LOC_Os02g42220 | 2 | 25392934 | 25399464 | 6530 | transposon protein, putative, unclassified, expressed | *OsYSL14(TM 12)* | NM_001054044 |
| 10 | LOC_Os02g43370 | 2 | 26163518 | 26170101 | 6583 | transposon protein, putative, unclassified, expressed | *OsYSL2(TM15)* | NM_001054121 |
| 11 | LOC_Os03g11010 | 3 | 5653093 | 5659215 | 6122 | natural resistance-associated macrophage protein, putative, expressed | *OsNramp2(TM10)* | NM_001055865 |
| 12 | LOC_Os03g19420 | 3 | 10924187 | 10927668 | 3481 | nicotianamine synthase, putative, expressed | *OsNAS2* | NM_001056429 |
| 13 | LOC_Os03g19427 | 3 | 10927201 | 10930613 | 3412 | nicotianamine synthase, putative, expressed | *OsNAS1* | NM_001056430 |
| 14 | LOC_Os03g41064 | 3 | 22821261 | 22824032 | 2771 | natural resistance-associated macrophage protein, putative, expressed | *OsNramp6(TM4)* | NM_001057173 |
| 15 | LOC_Os03g46460 | 3 | 26275162 | 26281225 | 6063 | ubiquitin carboxyl-terminal hydrolase, family 1, putative, expressed | *OsUCHL1* | NM_001057387 |
| 16 | LOC_Os03g46470 | 3 | 26278204 | 26286071 | 7867 | metal cation transporter, putative, expressed | *OsIRT1(TM9)* | NM_001057388 |
| 17 | LOC_Os04g32050 | 4 | 19047842 | 19054417 | 6575 | transposon protein, putative, unclassified, expressed | *OsYSL6(TM 14)* | NM_001059175 |
| 18 | LOC_Os04g32060 | 4 | 19054306 | 19060918 | 6612 | transposon protein, putative, unclassified, expressed | *OsYSL5(TM 9)* | NM_001059176 |
| 19 | LOC_Os04g36720 | 4 | 21996480 | 22002824 | 6344 | ferric-chelate reductase, putative, expressed | *OsFRO1(TM10)* | NM_001059431 |
| 20 | LOC_Os04g44290 | 4 | 26046729 | 26053380 | 6651 | expressed protein | expressed protein | NM_001059884 |
| 21 | LOC_Os04g44300 | 4 | 26050090 | 26056525 | 6435 | transposon protein, putative, unclassified, expressed | *OsYSL13(TM13)* | NM_001059885 |
| 22 | LOC_Os04g44320 | 4 | 26061602 | 26068144 | 6542 | transposon protein, putative, unclassified, expressed | *OsYSL12(TM12)* | NM_001059886 |
| 23 | LOC_Os04g45900 | 4 | 26997742 | 27003739 | 5997 | transposon protein, putative, unclassified, expressed | *OsYSL16(TM14)* | NM_001059985 |
| 24 | LOC_Os04g48930 | 4 | 28992724 | 28997525 | 4801 | ferric-chelate reductase, putative, expressed | *OsFRO2(TM6)* | NM_001060176 |
| 25 | LOC_Os04g52310 | 4 | 30892089 | 30896447 | 4358 | metal cation transporter, putative, expressed | *OsZIP3(TM6)* | NM_001060386 |
| 26 | LOC_Os05g07210 | 5 | 3806951 | 3811783 | 4832 | metal cation transporter, putative, expressed | *OsZIP6(TM8)* | NM_001061277 |
| 27 | LOC_Os05g10940 | 5 | 6089740 | 6095081 | 5341 | metal cation transporter, putative, expressed | *OsZIP7(TM6)* | NM_001061410 |
| 28 | LOC_Os05g39560 | 5 | 23152854 | 23157724 | 4870 | metal cation transporter, putative, expressed | *OsZIP5(TM7)* | NM_001062353 |

| **No.** | **gene locus name** | **Chr** | **Start** | **End** | **Region of Interest ROI (bp)** | **Description** | **Gene Symbol** | **accession number** |
| --- | --- | --- | --- | --- | --- | --- | --- | --- |
| 29 | LOC_Os06g29180 | 6 | 16647546 | 16653298 | 5752 | erythronate-4-phosphate dehydrogenase domain containing protein, expressed | *OsEry-4-PDH(FDH)* | NM_001064201 |
| 30 | LOC_Os06g37010 | 6 | 21822378 | 21826738 | 4360 | metal cation transporter, putative, expressed | *OsZIP10(TM5)* | NM_001187922 |
| 31 | LOC_Os07g15370 | 7 | 8869442 | 8878911 | 9469 | metal transporter Nramp6, putative, expressed | *OsNramp5(TM12)* | NM_001065847 |
| 32 | LOC_Os07g15460 | 7 | 8964029 | 8970888 | 6859 | metal transporter Nramp6, putative, expressed | *OsNramp1(TM11)* | NM_001065850 |
| 33 | LOC_Os08g10630 | 8 | 6265828 | 6270910 | 5082 | metal cation transporter, putative, expressed | *OsZIP4(TM7)* | NM_001067780 |
| 34 | LOC_Os08g17830 | 8 | 10936449 | 10945138 | 8689 | transposon protein, putative, unclassified, expressed | *OsYSL17(TM12)* | NM_001067977 |
| 35 | LOC_Os08g42150 | 8 | 26636759 | 26639353 | 2594 | zinc transporter 2 precursor, putative | *OsZIP2(TM3)* | NM_001052075 |
| 36 | LOC_Os10g38489 | 10 | 20481143 | 20490269 | 9126 | glutathione S-transferase GSTU6, putative, expressed | *OsGSTU6* | NM_001071659 |
| 37 | LOC_Os11g01530 | 11 | 302969 | 307933 | 4964 | ferritin-1, chloroplast precursor, putative, expressed | *OsFER1* | NM_001072072 |
| 38 | LOC_Os11g08210 | 11 | 4294051 | 4298685 | 4634 | no apical meristem protein, putative, expressed(transcription factor) | *OsNAC* | NM_001072451 |
| 39 | LOC_Os12g01530 | 12 | 318214 | 323923 | 5709 | ferritin-1, chloroplast precursor, putative, expressed | *OsFER1* | NM_001072468 |
| 40 | LOC_Os12g39180 | 12 | 24085315 | 24091178 | 5863 | natural resistance-associated macrophage protein, putative, expressed | *OsNramp7(TM11)* | NM_001073667 |
|  |  |  |  | **Total** | **240,225** |  |  |  |

**Supplementary table S3:** (cont.)
